# *Actinobacillus pleuropneumoniae* induces SJPL cell cycle arrest in G2/M-phase and inhibits porcine reproductive and respiratory syndrome virus replication

**DOI:** 10.1186/s12985-015-0404-3

**Published:** 2015-11-14

**Authors:** Jérémy A. Ferreira Barbosa, Josée Labrie, Francis Beaudry, Carl A. Gagnon, Mario Jacques

**Affiliations:** Centre de recherche en infectiologie porcine et avicole (CRIPA), Faculté de médecine vétérinaire, Université de Montréal, St-Hyacinthe, Québec Canada; Groupe de recherche sur les maladies infectieuses du porc (GREMIP), Faculté de médecine vétérinaire, Université de Montréal, St-Hyacinthe, Québec Canada; Groupe de recherche en pharmacologie animale du Québec (GREPAQ), Faculté de médecine vétérinaire, Université de Montréal, St-Hyacinthe, Québec, Canada

**Keywords:** *Actinobacillus pleuropneumoniae*, PRRSV, Antiviral effect, Host-pathogen interaction, Cell cycle

## Abstract

**Background:**

Porcine reproductive and respiratory syndrome virus (PRRSV) is one of the most important pathogens in the swine industry and causes important economic losses. No effective antiviral drugs against it are commercially available. We recently reported that the culture supernatant of *Actinobacillus pleuropneumoniae*, the porcine pleuropneumonia causative agent, has an antiviral activity *in vitro* against PRRSV in SJPL cells. Objectives of this study were (i) to identify the mechanism behind the antiviral activity displayed by *A. pleuropneumoniae* and (ii) to characterize the active molecules present in the bacterial culture supernatant.

**Methods:**

Antibody microarray analysis was used in order to point out cellular pathways modulated by the *A. pleuropneumoniae* supernatant. Subsequent, flow cytometry analysis and cell cycle inhibitors were used to confirm antibody microarray data and to link them to the antiviral activity of the *A. pleuropneumoniae* supernatant. Finally, *A. pleuropneumoniae* supernatant characterization was partially achieved using mass spectrometry.

**Results:**

Using antibody microarray, we observed modulations in G2/M-phase cell cycle regulation pathway when SJPL cells were treated with *A. pleuropneumoniae* culture supernatant. These modulations were confirmed by a cell cycle arrest at the G2/M-phase when cells were treated with the *A. pleuropneumoniae* culture supernatant. Furthermore, two G2/M-phase cell cycle inhibitors demonstrated the ability to inhibit PRRSV infection, indicating a potential key role for PRRSV infection. Finally, mass spectrometry lead to identify two molecules (m/z 515.2 and m/z 663.6) present only in the culture supernatant.

**Conclusions:**

We demonstrated for the first time that *A. pleuropneumoniae* is able to disrupt SJPL cell cycle resulting in inhibitory activity against PRRSV. Furthermore, two putative molecules were identified from the culture supernatant. This study highlighted the cell cycle importance for PRRSV and will allow the development of new prophylactic or therapeutic approaches against PRRSV.

**Electronic supplementary material:**

The online version of this article (doi:10.1186/s12985-015-0404-3) contains supplementary material, which is available to authorized users.

## Background

Coinfections are likely more frequent in farm than reported and it is well established that a primary infection with a pathogen, viral or bacterial, may enhance the infectious potential of a secondary pathogen [1–4]. The porcine respiratory disease complex (PRDC) is a multifactorial disease affecting fattening pigs that is caused by coinfections with viral and/or bacterial pathogens including the porcine reproductive and respiratory syndrome virus (PRRSV), *Actinobacillus pleuropneumoniae* and others [2, 5, 6]. PRDC is the most common disease in swine industry resulting in significant economic losses and is characterized by several symptoms including respiratory distress, fever, lethargy, stunted growth and death [2, 5, 6].

Coinfections are often studied by observing clinical symptoms in model animals; however, the basic mechanisms involved in these pathogen-pathogen interactions are often overlooked. *In vitro* investigations can provide insights for understanding coinfections. Our laboratory recently developed a model to study co-infections by *A. pleuropneumoniae* and PRRSV using SJPL cells [7].

PRRSV is a member of the *Arteriviridae* family and *Nidovirales* order. It is an enveloped, single-stranded positive sense RNA virus [8, 9]. The genome is approximately 15 kb in length and contains 11 open reading frames (ORF) [10–12]. PRRSV can infect pigs and trigger several symptoms (i.e. fever, inappetence, cyanosis), reproductive disorders (i.e. abortion, stillborn piglets, mummified fetuses) and respiratory disorders (i.e. cough, hyperpnea, dyspnea) [13–15]. Furthermore, PRRSV is the most important pathogen in swine production, causes important economic losses, and no effective antiviral drugs against it are commercially available [16].

*Actinobacillus pleuropneumoniae* (App) is the causative agent of porcine pleuropneumonia, an important disease in swine industry. The disease is well controlled in USA and Canada but still a significant problem in Latin America and some Asian and European countries [17]. *Actinobacillus pleuropneumoniae* is a Gram-negative rod-shaped bacteria and member of the *Pasteurellaceae* family. This bacterium is known to possess many virulence factors including lipopolysaccharides, capsular polysaccharides, outer membrane proteins involved in the acquisition of essential nutrients, surface molecules involved in adherence to the respiratory tract and Apx toxins [18]. For a recent review about virulence factors of *A. pleuropneumoniae* see Chiers and collaborators [18].

We recently reported that *A. pleuropneumoniae* culture supernatant has an antiviral activity against PRRSV *in vitro* in SJPL infected cells and in porcine alveolar macrophages [7]. This antiviral activity is not induced by *A. pleuropneumoniae* lipopolysaccharides or by peptidoglycan fragments (i.e. NOD1 and NOD2 ligands) [7]. The identity of the molecules responsible for the antiviral activity are unknown and their identification could provide the basis for the development of new therapeutic drugs, including prophylactic drugs with suitable biopharmaceutical properties against PRRSV infection. It is of note that experiments performed with culture supernatant of *Haemophilus parasuis* (strain Nagasaki), a close relative of *A. pleuropneumoniae*, did not show any antiviral activity against PRRSV (J. Labrie and M. Jacques, unpublished data).

We hypothesize that the culture supernatant of *A. pleuropneumoniae* induces a specific SJPL cell response which has an antiviral activity against PRRSV. The first objective of the present study was to identify the mechanism behind the antiviral activity displayed by *A. pleuropneumoniae*. The second objective was to identify the molecules present in the *A. pleuropneumoniae* culture supernatant which are responsible for the antiviral activity against PRRSV. Therefore, we first used an antibody microarray to identify cell pathways modulated by the *A. pleuropneumoniae* culture supernatant, observed modulations in cell cycle regulation pathways and confirm these modulations by cell cycle analysis using flow cytometry. We also demonstrated the ability of two known cell cycle inhibitors to inhibit PRRSV. Finally, mass spectrometry was used to detect and identify two molecules present only in the culture supernatant of *A. pleuropneumoniae*.

## Results

### Protein profiling of SJPL cells

**Fig. 1:**
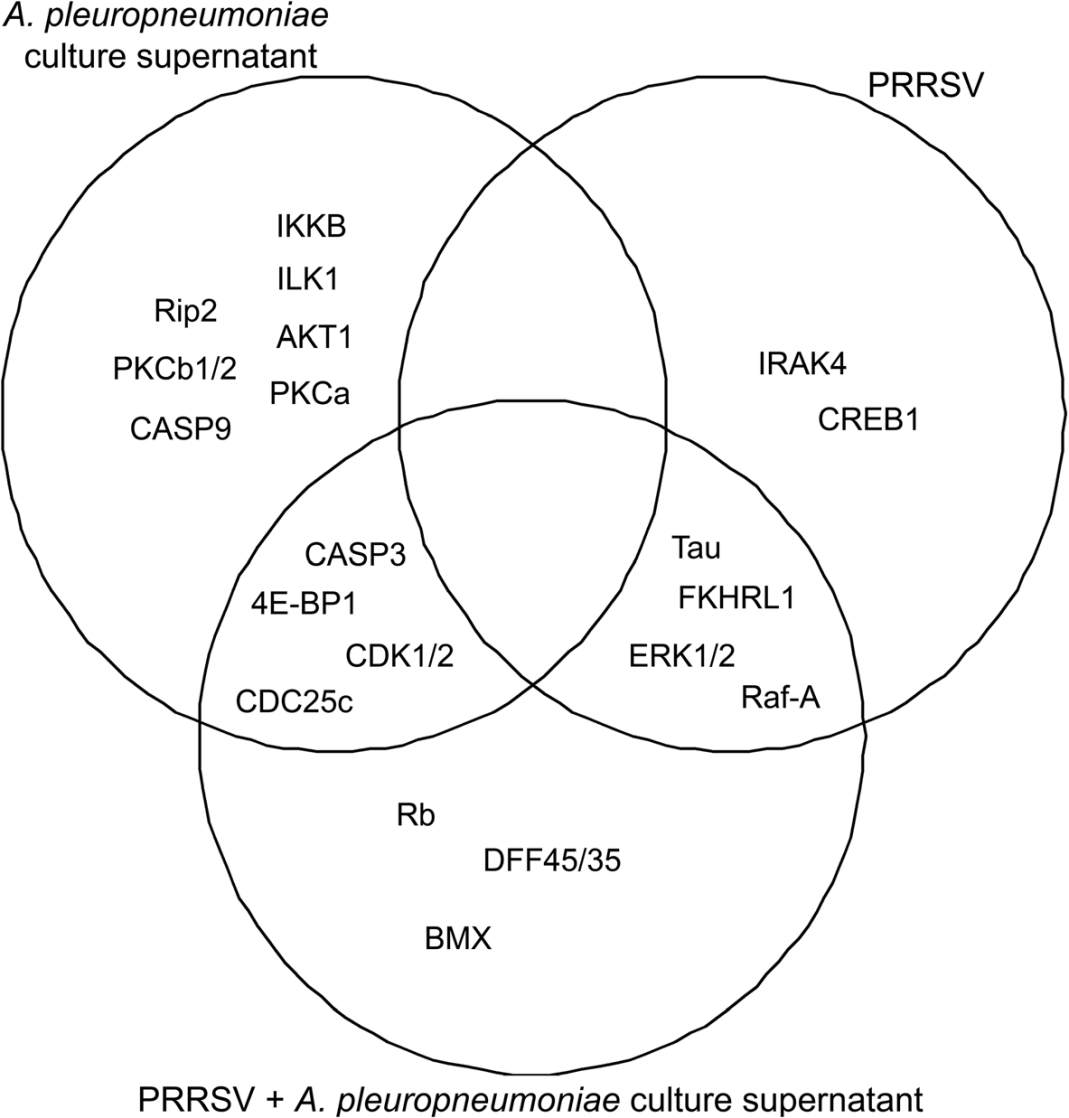
AppΔ*apxIC*Δ*apxIIC* culture supernatant upregulated cellular proteins when SJPL cells were infected or not with PRRSV. Proteins represented were selected using a Z-score difference (Z-score SJPL – Z-score SJPL treated/infected cells) threshold of plus 75 % (corresponding in log2 to 1.68 fold change versus the control). CDC25c: ser216 phosphorylated form; CDK1/2: tyr161 phosphorylated form; Tau: ser516 and ser519 phosphorylated forms; Rb: ser807 and ser811 phosphorylated forms

Lévesque and collaborators recently demonstrated that both AppΔ*apxIC*Δ*apxIIC* culture supernatant and its ≤ 3 kDa ultrafiltrate have an antiviral activity *in vitro* against PRRSV [7]. Therefore, protein profiling of SJPL cells infected or not with PRRSV (MOI 0.5) and/or treated or not with the AppΔ*apxIC*Δ*apxIIC* culture supernatant was performed using Kinex KAM-850 antibody microarray. Eight hundred and fifty four cell signaling proteins were targeted, using 337 phosphosite-specific antibodies and 517 pan-specific antibodies. Pan-specific antibodies targeted both phosphorylated and unphosphorylated proteins forms. Proteins were classified into nine groups according to their cellular functions: (1) transcription and translation factors; (2) proteins implicated in signal transduction pathway; (3) proteins implicated in host-pathogen interaction or in immune response; (4) proteins implicated in stress response; (5) proteins implicated in cell spreading, cell migration, cell survival, cell growth, cell cycle and cell proliferation; (6) apoptosis signaling pathway; (7) cytoskeleton-associated proteins; (8) proteins implicated in cell biosynthesis and metabolism; (9) proteins implicated in other biological process or unclassifiable proteins. Data are expressed in percent of change from control (mock-infected and untreated SJPL cells; % CFC) and are presented in Fig. 1 and Additional file 1: Table S1. The AppΔ*apxIC*Δ*apxIIC* culture supernatant upregulates inhibitor of nuclear factor kappa-B kinase subunit beta (IKKβ), receptor-interacting serine/threonine-protein kinase 2 (Rip2) and caspase-9 (CASP9). In cells treated with AppΔ*apxIC*Δ*apxIIC* culture supernatant, cell cycle regulation proteins (M-phase inducer phosphatase 3 inactive form (CDC25c (ser216)) and cyclin-dependent kinase 1/2 active forms (CDK1/2 (tyr161))) and caspase-3 (CASP3) were modulated by the culture supernatant regardless of whether they were infected with PRRSV or not. Also, PRRSV seems to upregulate mitogen-activated protein kinase (MAPK)/mitogen activated protein kinase ERK pathway and NF-κB pathway. Indeed, serine/threonine-protein kinase A-Raf (Raf-A), ERK1/2, interleukin-1 receptor-associated kinase 4 (IRAK4) and cyclic AMP-responsive element-binding protein 1 (CREB1) were differentially modulated in presence or absence of culture supernatant. Thus, PRRSV upregulates MAPK/ERK and NF-κB pathways. The culture supernatant seems to decrease the PRRSV induced upregulation for MAPK/ERK and NF-κB pathways. Overall, the antibodies microarray data suggests that *A. pleuropneumoniae* culture supernatant modulates SJPL cell cycle.

### Effect of the AppΔ*apxIC*Δ*apxIIC *culture supernatant on cell cycle

**Fig. 2:**
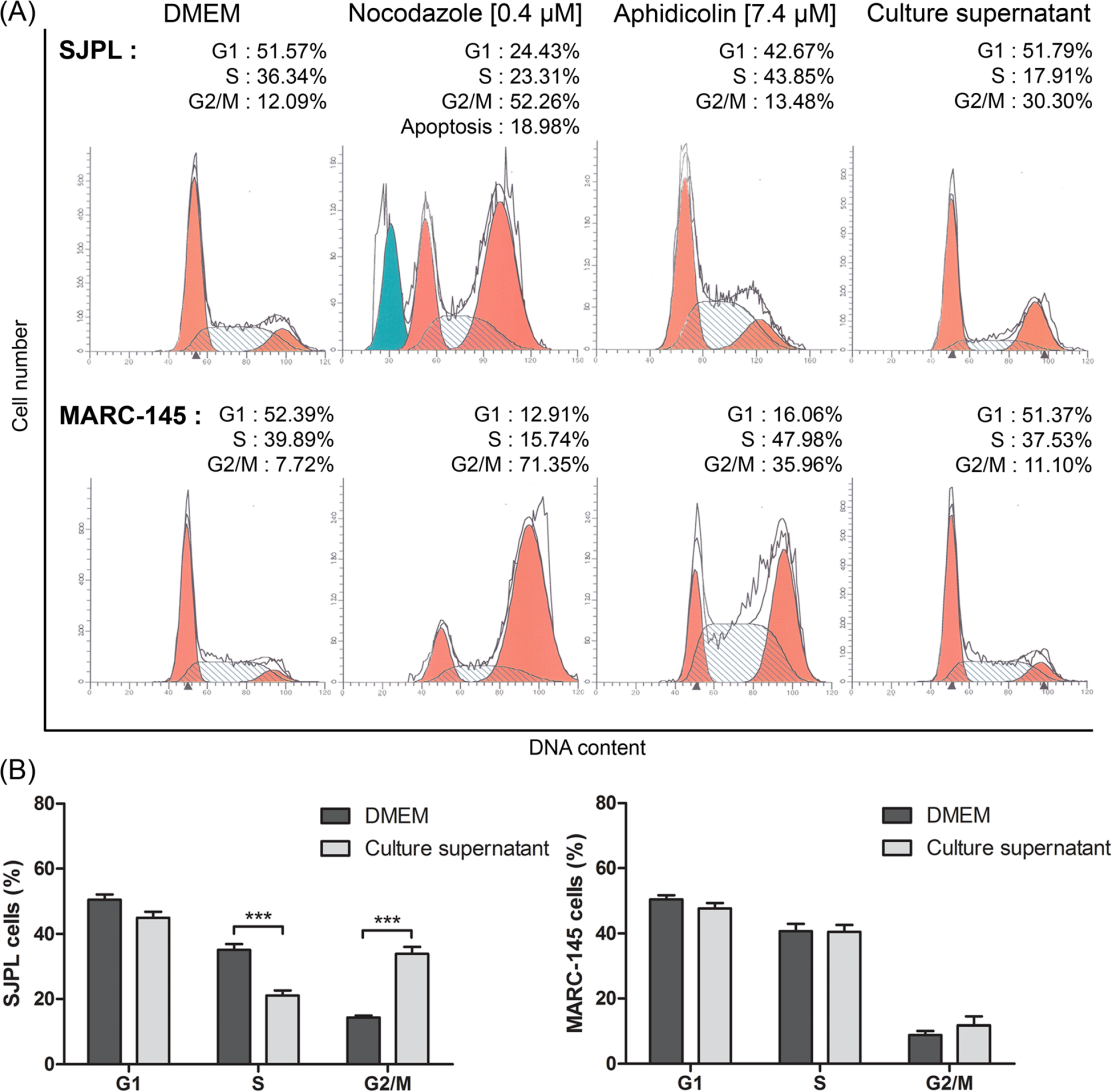
AppΔ*apxIC*Δ*apxIIC* culture supernatant blocks SJPL cell cycle in G2/M-phase but not MARC-145 cell cycle. **a** Representative experiment of SJPL and MARC-145 cell cycle profile analysis; DMEM, nocodazole and aphidicolin as controls are compared to the *A. pleuropneumoniae* culture supernatant. G1: Gap-1 phase; S: synthesis phase; G2/M: Gap-2 and mitotic phases. **b** Distribution of cell cycle phases after SJPL cells treatments with DMEM or AppΔ*apxIC*Δ*apxIIC* culture supernatant. The experiments were repeated at least 4 times. Asterisks indicated significant differences between untreated cells and cells treated with AppΔ*apxIC*Δ*apxIIC* culture supernatant. ***P ≤ 0.001, values are presented as ± standard error of the mean (SEM)

The microarray antibodies data suggesting that AppΔ*apxIC*Δ*apxIIC* culture supernatant modulates SJPL cell cycle, we decided to analyze SJPL and MARC-145 cell cycle after an 18 h treatment with the AppΔ*apxIC*Δ*apxIIC* culture supernatant, using flow cytometry. SJPL cells incubated in DMEM were used as normal cell cycle and we obtained a mean 50.6 % of SJPL cells in Gap-1 phase (G1-phase); 35.1 % of SJPL cells in DNA-synthesis phase (S-phase); and 14.3 % of SJPL cells in Gap-2 and mitotic phase (G2/M-phase) (Fig. 2a and b). We then used as controls, two molecules known to modulate the cell cycle at different phases. Nocodazole interferes with the polymerization of microtubules, blocking treated cells in G2/M-phase and inducing cell death by apoptosis [19]. Aphidicolin is a DNA-polymerase inhibitor and induces cell cycle arrest in S-phase [20]. When SJPL cells were treated with nocodazole or aphidicolin, we observed differences between treated and untreated cells (Fig. 2a). With nocodazole treatment, SJPL cell number in G2/M-phase increased (in mean) to 41.5% and an additional apoptotic peak before the G1-phase peak was seen as expected (Fig. 2a). With aphidicolin treatment, the proportion of SJPL cells in S-phase increased (in mean) to 51.9 % (Fig. 2a). Following validation with cell cycle controls, SJPL cells were treated with the AppΔ*apxIC*Δ*apxIIC* culture supernatant and significant variations in SJPL cell cycle proportions (*n* = 15; *P* ≤ 0.001) were observed. On average, proportion of SJPL cells in G1-phase stayed the same as untreated cells (45.0 %). However, the proportion of SJPL cells in S-phase and in G2/M-phase significantly (*n* = 15; *P* ≤ 0.001) decreased to 21.1 % and increased to 33.9 %, respectively (Fig. 2a and b), indicating that *A. pleuropneumoniae* culture supernatant had an effect on SJPL cell cycle.

Lévesque and collaborators reported that the antiviral effect of AppΔ*apxIC*Δ*apxIIC* culture supernatant against PRRSV was not observed in MARC-145 infected cells [7]. To provide supplemental insight of the impact of cell cycle arrests caused by the *A. pleuropneumoniae* culture supernatant on SJPL infected cells and on PRRSV infection, we treated MARC-145 cells with the AppΔ*apxIC*Δ*apxIIC* culture supernatant and analyzed their cell cycle. Cell cycle of untreated and uninfected MARC-145 cell was similar to the SJPL cell cycle. Proportion of MARC-145 cells in the three phases was: 50.5 % (G1-phase), 40.7 % (S-phase) and 8.8 % (G2/M-phase) (Fig. 2a and b). When cells were treated with nocodazole or aphidicolin, an increase of the proportion of MARC-145 cells in G2/M-phase for nocodazole or in S-phase and G2/M-phase for aphidicolin was observed (Fig. 2a). In contrast to SJPL cells, when MARC-145 cells were treated with the culture supernatant, no differences were observed between treated and control cells (Fig. 2a and b).

### Effect of the ≤ 3 kDa AppΔ*apxIC*Δ*apxIIC *culture supernatant ultrafiltrate on SJPL cell cycle

**Fig. 3:**
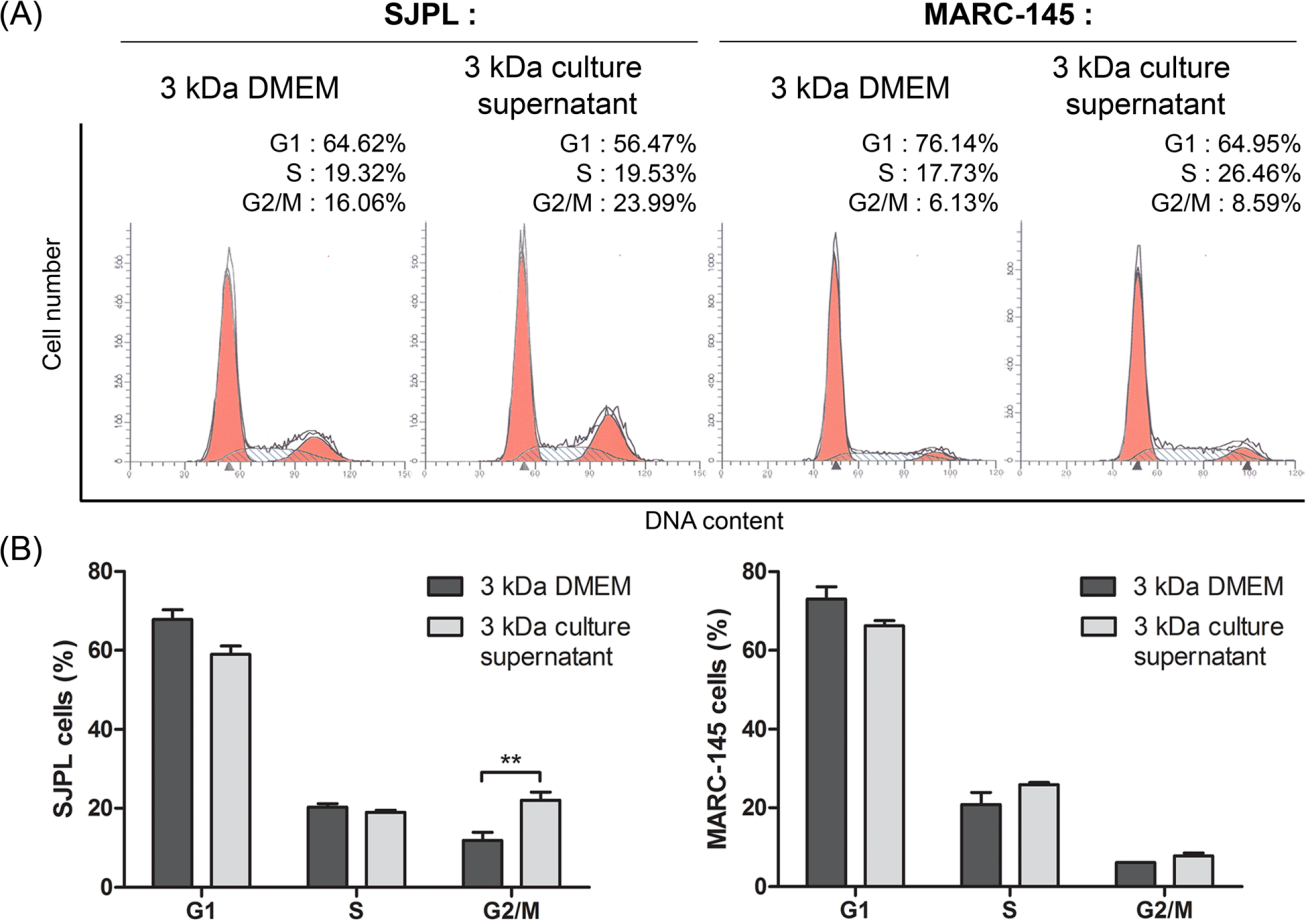
The ≤ 3 kDa AppΔ*apxIC*Δ*apxIIC* ultrafiltrate is sufficient to block SJPL cell cycle in G2/M-phase. **a** Representative experiment of SJPL and MARC-145 cell cycle profile analysis; ≤ 3 kDa DMEM ultrafiltrate as control is compared to the ≤ 3 kDa AppΔ*apxIC*Δ*apxIIC* culture supernatant ultrafiltrate. **b** Distribution of cell cycle phases after SJPL cells treatments with the ≤ 3 kDa DMEM ultrafiltrate or the ≤ 3 kDa AppΔ*apxIC*Δ*apxIIC* culture supernatant ultrafiltrate. The experiments were repeated at least 2 times. Asterisks indicated significant differences. **P ≤ 0.01, values are presented as ± SEM

Knowing that AppΔ*apxIC*Δ*apxIIC* culture supernatant modulates SJPL cells cycle, we treated cells with the ≤ 3 kDa DMEM ultrafiltrate (as control) or the ≤ 3 kDa AppΔ*apxIC*Δ*apxIIC* culture supernatant ultrafiltrate to know if the ≤ 3 kDa fraction also modulates the SJPL cell cycle. The ≤ 3 kDa AppΔ*apxIC*Δ*apxIIC* culture supernatant ultrafiltrate significantly (*n* = 4; *P* ≤ 0.01) increased the number of cells in G2/M-phase from 11.8 % to 22.1 % (in mean) (Fig. 3a and b (left side)). Thus, the ≤ 3 kDa AppΔ*apxIC*Δ*apxIIC* culture supernatant ultrafiltrate which possesses the antiviral activity against PRRSV also modulates SJPL cell cycle. No modulation of the cell cycle was also observed in MARC-145 cells treated with the AppΔ*apxIC*Δ*apxIIC* culture supernatant (Fig. 3a and b right side). This suggests a potential link between cell cycle arrest in G2/M-phase and antiviral activity against PRRSV in SJPL cells.

### Effect of DIM and SBE-13 on SJPL cell cycle

**Fig. 4:**
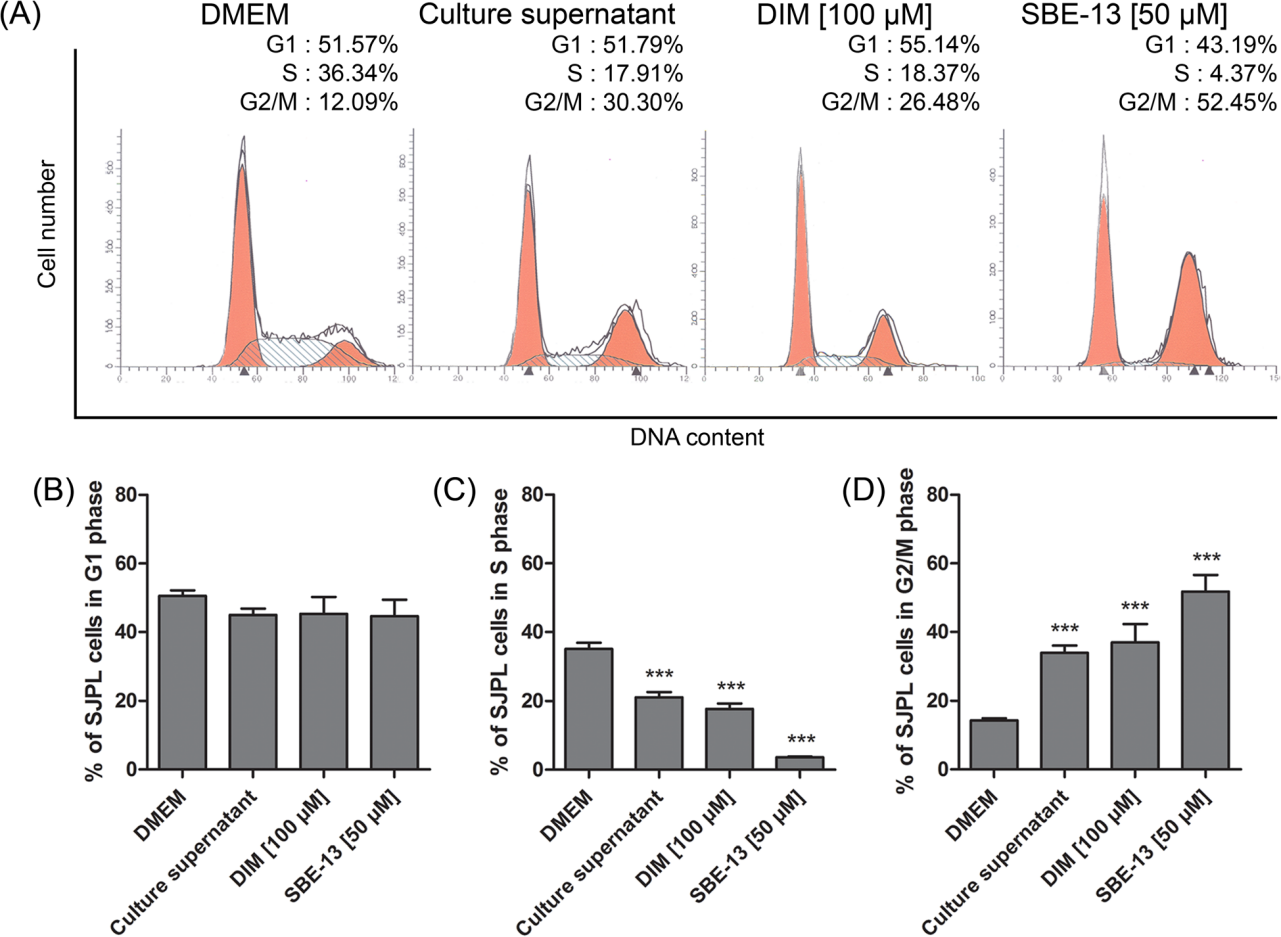
Chk2 activator and PLK inhibitor are both mimics of the AppΔ*apxIC*Δ*apxIIC* culture supernatant. **a** Representative experiment of SJPL cell cycle profile analysis; DMEM and AppΔ*apxIC*Δ*apxIIC* culture supernatant are compared to DIM (Chk2 activator) and SBE-13 (PLK inhibitor). **b** Percentage of cells in G1 phase. **c** Percentage of cells in S-phase. **d** Percentage of cells in G2/M-phases. The experiments were repeated at least 6 times. Asterisks indicated significant differences between DMEM cells and treated cells. ***P ≤ 0.001, values are presented as ± SEM

We have previously shown, using protein profiling, that AppΔ*apxIC*Δ*apxIIC* culture supernatant upregulates CDC25c (ser216) and CDK1/2 (tyr161), both of which are implicated in the G2/M cell cycle regulation pathway. Two specific cell cycle inhibitors were used to verify whether cell cycle modulation induced by the culture supernatant is due to CDC25c and/or CDK1/2. We first used DIM which is a specific activator of cell cycle inhibitor serine/threonine-protein kinase (Chk2), Chk2 is an inhibitor of CDC25 and leads to a cell cycle arrest in G2/M-phase [21]. Then, we used SBE-13 which is a selective inhibitor of cell cycle activator polo-kinase isoforms: PLK1, PLK2, PLK3 [22]. These proteins are implicated in the G2/M-phase transition pathway; PLK1 is a CDC25c activator and CDC25c promotes the G2/M-phase transition using cyclin-B/CDK1 complex. Both DIM and SBE-13 treatments induced a SJPL cell cycle modulation (Fig. 4). G1-phase of SJPL cells was not modulated by DIM or SBE-13 (Fig. 4a and b). The number of cells in S-phase was significantly (n ≥ 6; *P* ≤ 0.001) decreased (in mean) from 35.1 % to 17.7 % and to 3.6 % by DIM and SBE-13 treatments, respectively (Fig. 4a and c) and proportions of cells in G2/M-phase were significantly (n ≥ 6; *P* ≤ 0.001) increased (in mean) from 14.3 % to 37.0 % and to 51.8 % by DIM and SBE-13 treatments, respectively as previously observed with *A. pleuropneumoniae* culture supernatant (Fig. 4a and d).

### Evaluation of SJPL cell proliferation

**Fig. 5:**
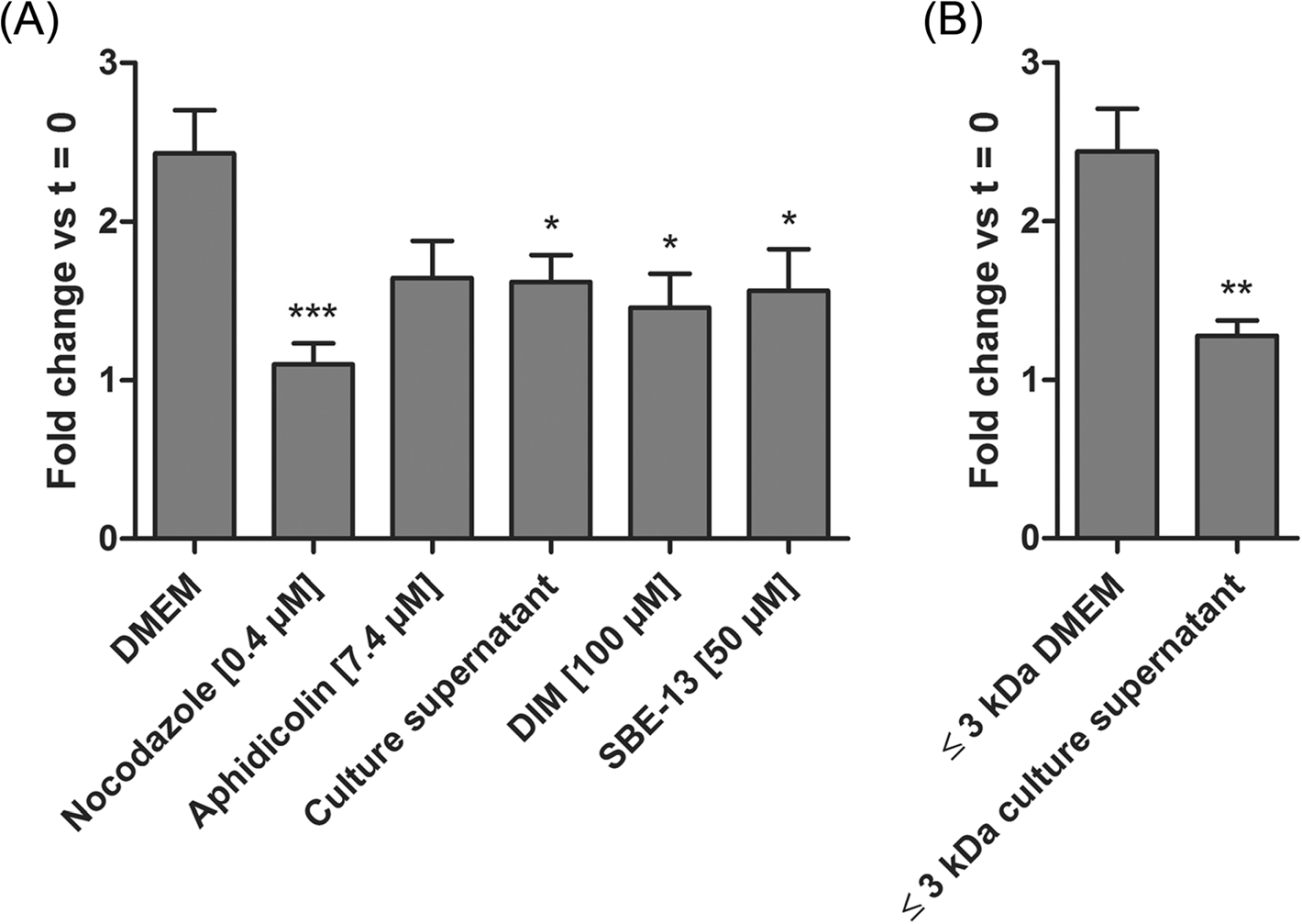
SJPL cell proliferation is inhibited by DIM and SBE-13. Cell counts were carried out before and after treatments as proliferation indicator. **a** Cells treated with nocodazole (known to induce apoptosis), aphidicolin, AppΔ*apxIC*Δ*apxIIC* culture supernatant, DIM or SBE-13. **b** Cells treated with the ≤ 3 kDa AppΔ*apxIC*Δ*apxIIC* culture supernatant ultrafiltrate is compared to the ≤ 3 kDa DMEM ultrafiltrate. The experiments were repeated at least 8 times. Asterisks indicated significant differences between DMEM cells or the ≤ 3 kDa DMEM ultrafiltrate and treated cells. *P ≤ 0.05, **P ≤ 0.01, ***P ≤ 0.001, values are presented as ± SEM

In order to confirm the cell cycle arrest we performed cell counts as an indicator of cell proliferation. Cells were counted before and after treatments and ratios between the second and the first counts were determined. These ratios were then compared to untreated cells ratio (DMEM) or to the ≤ 3 kDa DMEM ultrafiltrate ratio (Fig. 5a). We have previously shown that nocodazole and aphidicolin blocks SJPL cell cycle (Fig. 2a, upper panel) and decreases SJPL proliferation (Fig. 5a). Furthermore, cells treated with the AppΔ*apxIC*Δ*apxIIC* culture supernatant, DIM or SBE-13 showed significantly (*n* = 8; *P* ≤ 0.05) less proliferation than cells treated with DMEM. Same effect was observed between cells treated with the ≤ 3 kDa DMEM ultrafiltrate and cells treated with the ≤ 3 kDa AppΔ*apxIC*Δ*apxIIC* culture supernatant ultrafiltrate; from a fold change of 2.4 to 1.3 ( *n* = 8; *P* ≤ 0.01). These data indicated that the AppΔ*apxIC*Δ*apxIIC* culture supernatant or the ≤ 3 kDa AppΔ*apxIC*Δ*apxIIC* culture supernatant ultrafiltrate significantly decreased SJPL cells proliferation.

### Detection of PRRSV in SJPL infected and treated cells

**Fig. 6:**
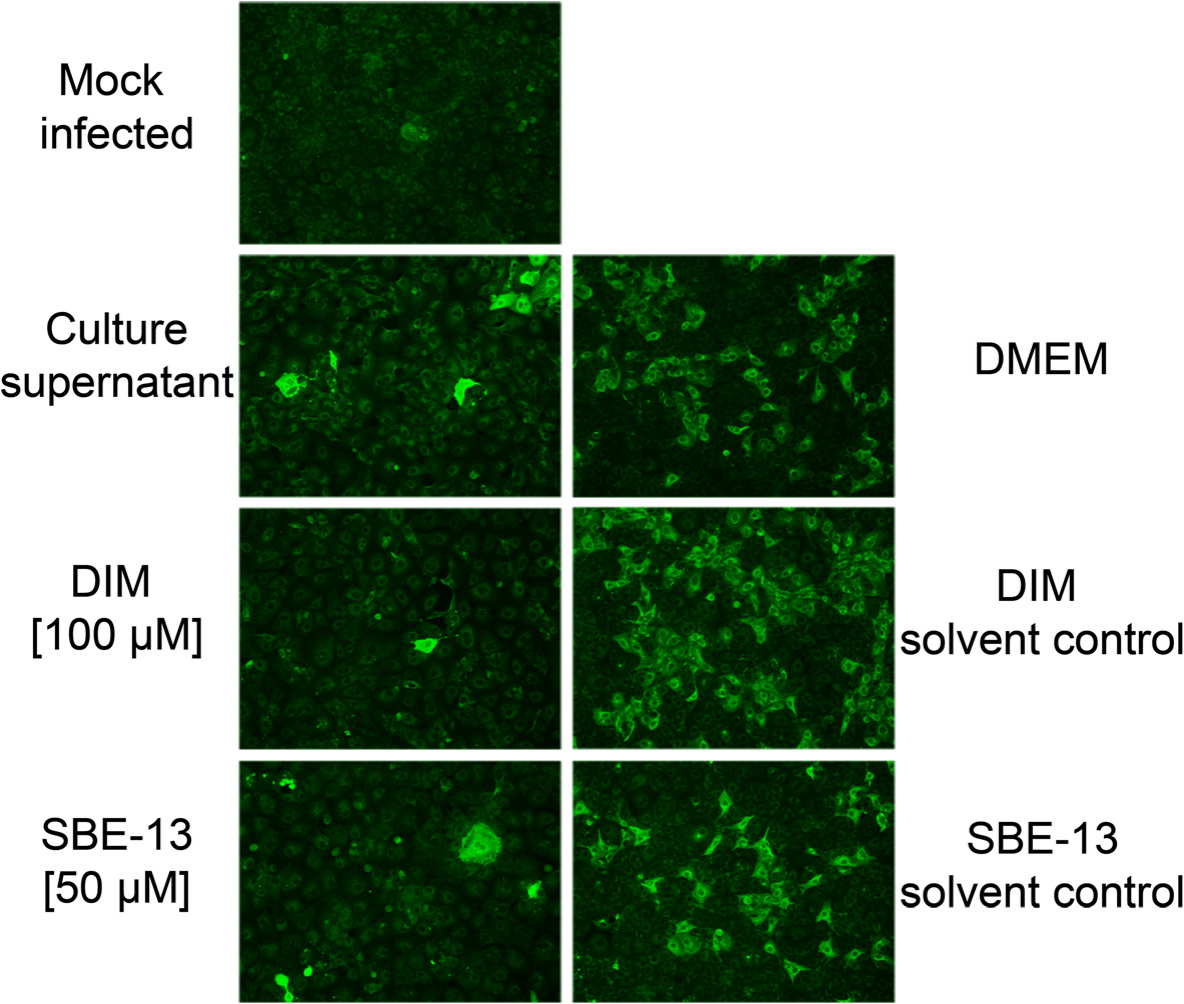
Detection of PRRSV in SJPL infected and treated cells. Primary antibodies targeted the PRRSV N protein and were revealed by an anti-rabbit antiserum FITC-conjugated antibody. Representative immunofluorescence pictures were acquired at 100 × magnification

Knowing that the culture supernatant disrupts SJPL cell cycle and decreases SJPL cell proliferation we were interested to see whether both cell cycle inhibitors, DIM and SBE-13, can also block PRRSV infection in SJPL cells. When SJPL cells were infected with PRRSV and treated with the AppΔ*apxIC*Δ*apxIIC* culture supernatant, an antiviral activity was observed (Fig. 6) as previously reported by Lévesque and collaborators [7]. When SJPL infected cells were treated with DIM, no PRRSV was detected; in contrast to DIM solvent control (Fig. 6). Similar observations can be done for cells treated with SBE-13 and SBE-13 solvent control (Fig. 6). Absence of PRRSV in SJPL infected cells treated with DIM and SBE-13 suggests an antiviral effect of these two cell cycle inhibitors against PRRSV.

### Mass spectrometry analysis

**Fig. 7:**
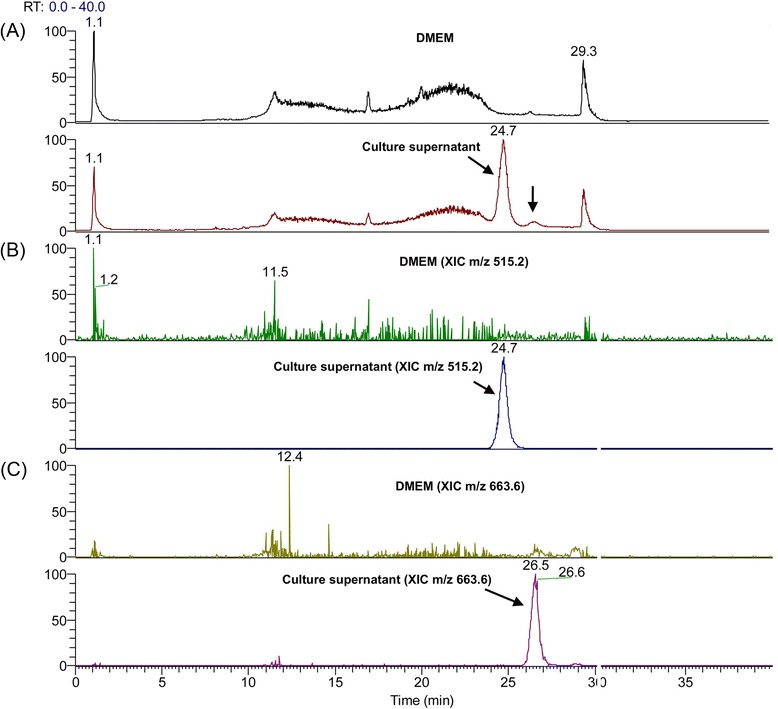
Differential comparison of ions present in DMEM or AppΔ*apxIC*Δ*apxIIC* culture supernatant using LC-MS/MS. Samples were analyzed by LC-MS/MS in full scan positive ion mode. **a** TIC of DMEM and AppΔ*apxIC*Δ*apxIIC* culture supernatant. **b** XIC m/z 515.2. **c** XIC m/z 663.6. Black arrows: additional peaks in the culture supernatant

In order to identify the actives molecules present in the AppΔ*apxIC*Δ*apxIIC* culture supernatant, mass spectrometry was used. Interestingly, mass spectrometry and collision-induced dissociation can provide comprehensive spectra allowing structural information to be derived with assistance of bioinformatics. Thus, mass spectrometry was used to analyze the ≤ 3 kDa AppΔ*apxIC*Δ*apxIIC* culture supernatant ultrafiltrate to detect, identify and characterize active molecules. The ≤ 3 kDa DMEM and ≤ 3 kDa AppΔ*apxIC*Δ*apxIIC* culture supernatant ultrafiltrates were fractionated using a simple salting-out liquid-liquid extraction in order to remove undesired inorganic molecules. Supernatant were then analyzed in full scan positive ion mode by LC-MS/MS and results are presented with a total ion current chromatogram (TIC) (Fig. 7a). Both ≤ 3 kDa DMEM ultrafiltrate and ≤ 3 kDa AppΔ*apxIC*Δ*apxIIC* culture supernatant ultrafiltrate were then compared and two unique peaks were observed in the ≤ 3 kDa AppΔ*apxIC*Δ*apxIIC* culture supernatant ultrafiltrate (Fig. 7a, black arrows). Full scan spectra and extracted ion chromatograms show the presence of two individual peaks at mass to charge (m/z) 515.2 (Fig. 7b) and m/z of 663.6 (Fig. 7c). Product ion spectra for these two peaks were acquired. At m/z 515.2, four ions were observed at m/z 160.6; 239.6; 328.6; 347.6, and six ions were observed for m/z 663.6 at m/z 495.0; 496.11; 551.0; 552.0; 606.9; and 607.9. However, using *in silico* fragmentation predictor and database search (e.g. METLIN) we did not succeed to unequivocally identify these two molecules present in *A. pleuropneumoniae* culture supernatant.

### Separation of the ≤ 3 kDa AppΔ*apxIC*Δ*apxIIC *culture supernatant ultrafiltrate using thin-layer chromatography

**Fig. 8:**
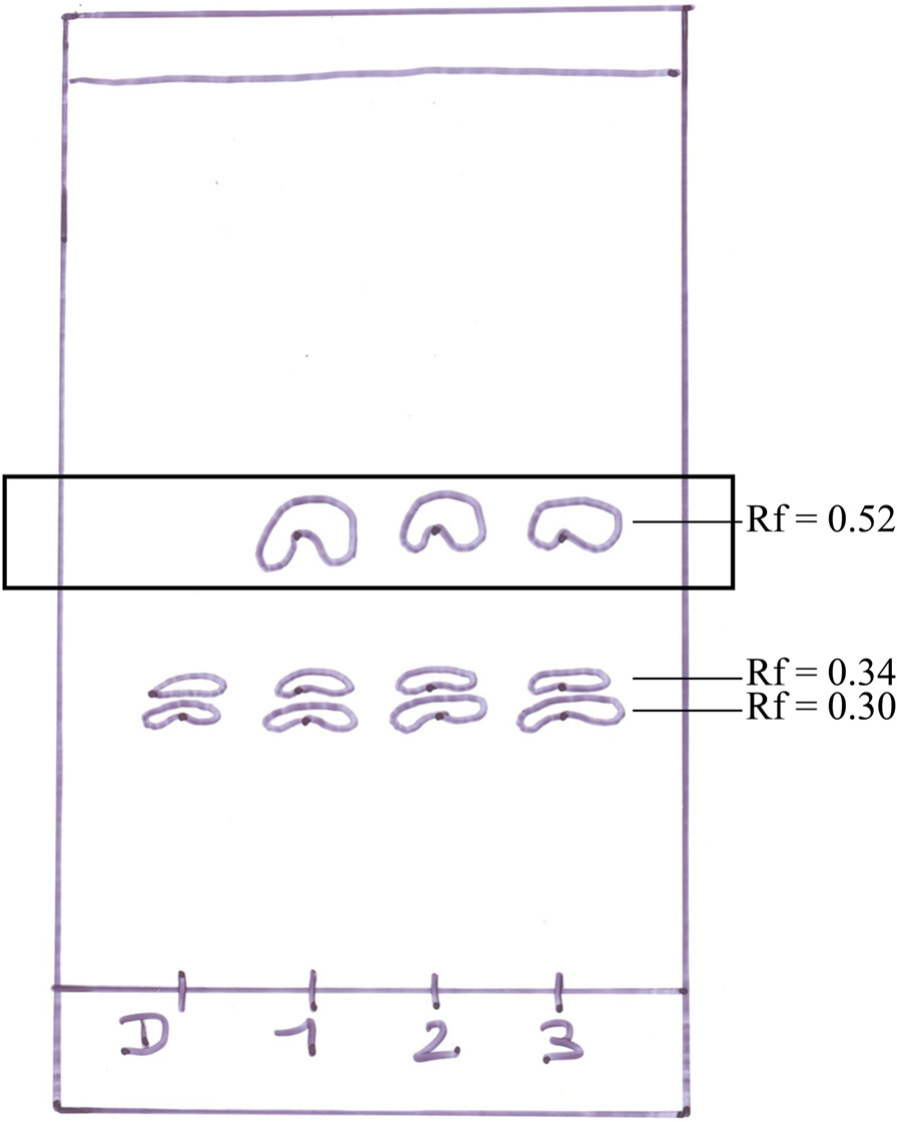
Migration of DMEM and culture supernatant of different strains of *A. pleuropneumoniae* by TLC. Representation of the TLC plate after migration and revelation under UV-A light. D: DMEM, as control; 1: AppΔ*apxIC*Δ*apxIIC* culture supernatant; 2: *A. pleuropneumoniae* (serotype 5b, strain L20) culture supernatant ; 3: *A. pleuropneumoniae* (serotype 1, strain 4074) culture supernatant. Framed spots: spots of interest (Rf = 0.52) that were analyzed by LC-MS/MS

**Fig. 9:**
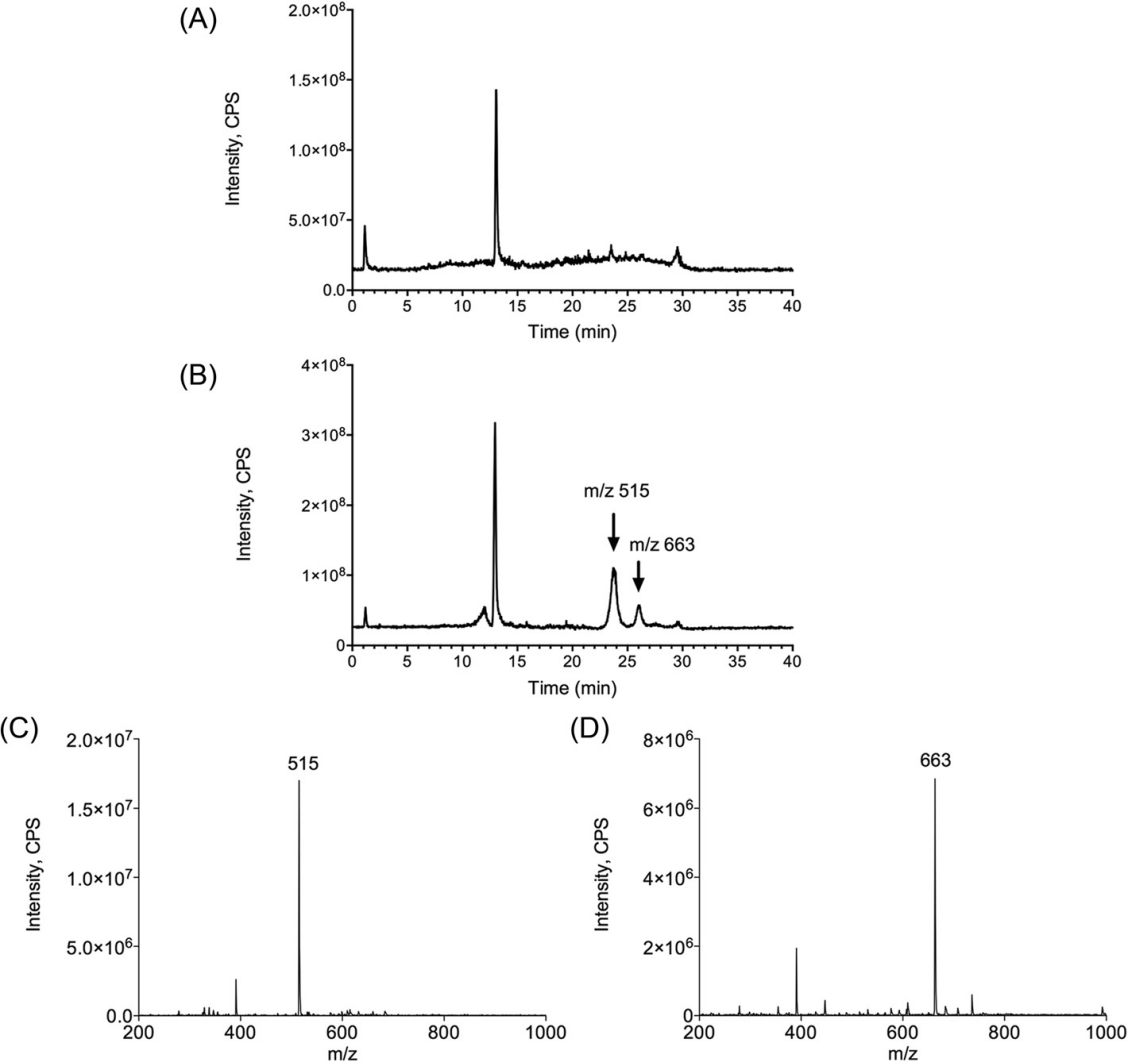
LC-MS/MS analyses of the molecules extracted from the TLC spot (Rf = 0.52). **a** TIC of the control TLC spot. **b** TIC of the TLC spot present in the AppΔ*apxIC*Δ*apxIIC* culture supernatant. **c** XIC m/z 515.2 of the AppΔ*apxIC*Δ*apxIIC* culture supernatant TLC spot. **d** XIC m/z 663.6 of the AppΔ*apxIC*Δ*apxIIC* culture supernatant TLC spot. Unique and signature peaks at m/z 515.2 and 663.6 were observed exclusively into AppΔ*apxIC*Δ*apxIIC* culture supernatant

In order to improve fractionation of the ≤ 3 kDa AppΔ*apxIC*Δ*apxIIC* culture supernatant ultrafiltrate and test molecules activities, we separated the suspected active ingredients using thin-layer chromatography and compare results with standards run on the same chromatogram. Migrations were carried out using a chloroform/methanol mixture and were then read using UV-A. We observed a distinctive spot (retention factor (Rf) = 0.52) present in the ≤ 3 kDa AppΔ*apxIC*Δ*apxIIC* culture supernatant ultrafiltrate (Fig. 8). Interestingly, this distinctive spot was also present in the ≤ 3 kDa culture supernatant ultrafiltrate of two reference strains of *A. pleuropneumoniae*: L20 (serotype 5b) and S4074 (serotype 1) (Fig. 8). TLC spot (Rf = 0.52) was excised and molecules were extracted with acetonitrile and then analyzed by LC-MS/MS. TIC and extracted ion chromatogram (XIC) of the ≤ 3 kDa AppΔ*apxIC*Δ*apxIIC* culture supernatant ultrafiltrate TLC spot (Rf = 0.52) possesses both unique peaks observed at m/z 515.2 and m/z 663.6 (Fig. 9). Thus, peaks corresponding to m/z 515.2 and 663.6 correspond to the additional TLC spot present in the *A. pleuropneumoniae* culture supernatant.

## Discussion

Lévesque and collaborators recently reported an *in vitro* antiviral activity of the *A. pleuropneumoniae* culture supernatant against PRRSV in SJPL infected cells and in porcine alveolar macrophages [7]. The objective of the study presented here was to identify the mechanism behind the antiviral activity displayed by *A. pleuropneumoniae* and to identify the active molecules present in *A. pleuropneumoniae* culture supernatant.

In SJPL infected cells, we demonstrated that PRRSV strongly upregulates mitogen-activated protein kinase (MAPK)/ERK pathway using ERK1/2, RAF-A and CREB. Our results are in agreement with Lee and Lee which demonstrated an activation of ERK1/2 during PRRSV infection [23] and with Han and collaborators which highlighted that PRRSV induces degradation of CREB-binding protein (CBP) [24]. In contrast, treatment of cells with *A. pleuropneumoniae* culture supernatant decreased the upregulation of CREB1 and ERK1/2 induced by PRRSV demonstrating that the *A. pleuropneumoniae* culture supernatant decrease MAPK/ERK and NF-κB pathways upregulation induced by PRRSV. This observation is interesting as it can partly explain the antiviral activity of the culture supernatant against PRRSV and should be further investigated.

Peptidoglycan fragments can stimulate the nucleotide-binding oligomerization domain (NOD) pathway [25, 26]. Following activation, NOD1 or NOD2 recruits RIP2 and nuclear factor of kappa light polypeptide gene enhancer in B-cells inhibitor alpha (IκBα), leading to NF-κB activation [27]. Protein profiling indicated that only RIP2, a specific NF-κB activator, was upregulated by the culture supernatant in absence of PRRSV. The absence of NF-κB activation by *A. pleuropneumoniae* culture supernatant alone is certainly puzzling, but Lévesque and collaborators demonstrated that NOD pathway induction using peptidoglycan fragments did not lead to an antiviral effect [7]. Our results confirm their observation.

Auger and collaborators demonstrated a differential expression of NF-κB pathway in SJPL cells after 3 h of infection with *A. pleuropneumoniae* serotype 1 (S4074) [28]. In SJPL cells treated with *A. pleuropneumoniae* culture supernatant, we only observed a weak upregulation of IKKβ. Auger and collaborators implicated the *A. pleuropneumoniae* lipopolysaccharides in NF-κB pathway activation [28]. Absence of NF-κB activation by the culture supernatant cannot be explained because *A. pleuropneumoniae* lipopolysaccharides were not removed from the culture supernatant. Conversely, PRRSV seems to be able to activate the NF-κB pathway in SJPL cells, as was already observed in MARC-145 and alveolar macrophages [29].

Apoptosis is an important cell process which can be regulated by caspases proteins [30]. We observed that *A. pleuropneumoniae* culture supernatant weakly regulates CASP3 and CASP9. Apx toxins from *A. pleuropneumoniae* are known to induce apoptosis through activation of c-Jun N-terminal kinase (JNK)/MAPK pathway and CASP3 [31, 32], as using an Apx mutant prevent this induction of JNK/MAPK pathway. Our results suggest that *A. pleuropneumoniae* culture supernatant do not induce apoptosis. We did not observe any peak corresponding to apoptosis during cell cycle analysis (Figs. 2a and 3a). These results were in agreement with Auger and collaborator, who carried out Western blot to detect CASP3 activation and concluded that *A. pleuropneumoniae* was not inducing apoptosis in SJPL cells [28].

Cell proliferation is a tightly controlled process. Many cell cycle checkpoints can be modulated to provoke cell cycle arrest. The G2/M-phase checkpoint is activated after DNA damages and provides opportunity to stop and repair the damaged cells. To the best of our knowledge, the observed cell cycle arrest induced by *A. pleuropneumoniae* has never been reported before. However, *Aggregatibacter actinomycetemcomitans* (formerly *Actinobacillus actinomycetemcomitans*) was able to stop growth and induce cell cycle arrest in G2/M-phase of HeLa cells, by production of the cytolethal distending toxin (CDT) [33, 34]. With regards to PRRSV, Sun and collaborators demonstrated the ability of its non structural protein 11 to control the cell cycle and to induce a cell cycle arrest in S-phase [35]. We demonstrated that *A. pleuropneumoniae* culture supernatant is able to induce a G2/M-phase cell cycle arrest in SJPL cells, resulting in decreased cell proliferation. Using CDC25c, CDK1/2 and PLK inhibitors, we confirmed the protein profiling results. DIM and SBE-13 are known to induce a cell cycle arrest in G2/M-phase and both decreased proliferation of SJPL cells. The cell cycle arrest induced by *A. pleuropneumoniae* can be due to a CDC25c or CDK1/2 inhibition. We will further investigate this pathway using Western-blot analysis of regulation proteins.

We also studied the effect of the culture supernatant on the MARC-145 cell line. Lévesque and collaborators have shown that the culture supernatant of *A. pleuropneumoniae* has no antiviral effect in MARC-145 cells [7]. In agreement with this finding, our cell cycle observations demonstrated a differentiated effect of the culture supernatant on SJPL and on MARC-145 cells. In SJPL cells, the culture supernatant induced a cell cycle arrest in G2/M-phase, whereas the same culture supernatant has no antiviral and no cell cycle effects on MARC-145 cells. Similar effects were observed for cell proliferation and for the cell cycle arrest induce by DIM and SBE-13. These differences between SJPL and MARC-145 cells support our results indicating that the culture supernatant inhibited PRRSV in SJPL cells by inducing a G2/M-phase cell cycle arrest.

Using tandem mass spectrometry we have isolated and identified two unique peaks (m/z 515 and m/z 663) from the ≤ 3 kDa AppΔ*apxIC*Δ*apxIIC* culture supernatant ultrafiltrate. We were able to separate and purify the ≤ 3 kDa AppΔ*apxIC*Δ*apxIIC* culture supernatant ultrafiltrate using TLC plates, and more interestingly, to recover the two peaks after TLC spots extraction. Unfortunately, mass spectrometry fingerprinting did not lead to identification of these molecules corresponding to these two peaks. In order to obtain more information and to definitely identify active molecules, we have to refine extraction protocols and to obtain higher purity products in amounts compatible with ^1^H and ^13^C nuclei nuclear magnetic resonance (NMR).

## Conclusions

We have demonstrated for the first time that *A. pleuropneumoniae* disrupts the SJPL cell cycle and prevents SJPL cell proliferation. We have also demonstrated the importance of cell cycle in PRRSV infection. Furthermore, two putative molecules were identified from the culture supernatant. It seems likely that these molecules are responsible for the antiviral activity of *A. pleuropneumoniae* against PRRSV. We have to further investigate these molecules in the hope that it will lead to the development of a new antiviral drug against PRRSV.

## Methods

### Cell lines

All cell culture products were purchased from Gibco (Life Technologies, Grand Island, NY, USA). SJPL cell line [36] and MARC-145 cell line [37] are known to support PRRSV replication [38, 39]. SJPL cells were provided by R.G. Webster (St. Jude Children’s Hospital, Memphis, TN, USA) and were historically defined as porcine lung cells [36] but were later found to come from monkey [40]. SJPL cells were grown in Dulbecco’s modified Eagle’s medium (DMEM) supplemented with 10 % (v/v) fetal bovine serum (FBS), 2 mM L-glutamine, 1 mM sodium pyruvate, 1 % (v/v) MEM non-essential amino acid (NEAA) solution, 100 μg/mL streptomycin, 100 U/mL penicillin, 50 mg/L gentamicin and 2.5 mg/L amphotericin B. MARC-145 cell line is a subclone of African green monkey kidney cell line MA104 [38]. MARC-145 cells were grown in minimum essential medium (MEM) supplemented with 10 % (v/v) FBS, 2 mM L-glutamine, 10 mM HEPES, 10 μg/mL streptomycin, 10 U/mL penicillin and 2.5 mg/L amphotericin B. Cells were cultured and infected at 37 °C in a 5 % CO_2_ atmosphere.

### Bacterial and viral strains

*Actinobacillus pleuropneumoniae* non-hemolytic and non-cytotoxic MBHPP147 was kindly provided by Ruud P.A.M. Segers (MSD Animal Health, Boxmeer, The Netherlands). This strain is a mutant of the serotype 1 reference strain S4074 producing non-active ApxI and ApxII toxins (AppΔ*apxIC*Δ*apxIIC*) [41]. Bacteria was grown at 37°C in brain heart infusion broth (BHI; Oxoid Ltd., Basingstoke, Hampshire, England) or in BHI agar (Oxoid Ltd.) supplemented with 5 μg/mL or 15 μg/mL of β-nicotinamide adenine dinucleotide (β-NAD; Sigma-Aldrich, St Louis, MO, USA), respectively.

The PRRSV North American reference strain IAF-Klop was used in this study. This strain is a genotype II strain [42, 43].

### Bacterial culture supernatant

*Actinobacillus pleuropneumoniae* culture supernatant was prepared as recently described by Lévesque and collaborators [7]. Briefly, an overnight culture of AppΔ*apxIC*Δ*apxIIC* was diluted into fresh BHI broth and grown to an OD_600_ of 0.6. This culture was then diluted into DMEM medium, supplemented with 10 % FBS, 2 mM L-glutamine, 1 mM sodium pyruvate, 1 % NEAA, to 1 × 10^6^ CFU/mL and incubated overnight at 37 °C. Bacterial cells were centrifuged at 4,000 *g* for 20 min and the supernatant was harvested and passed through a 0.22 μm filter (Sarstedt Inc., Newton, NC, USA). Filter-sterilized supernatant was ultrafiltrated through a 3 kDa membrane (Merck Millipore Ltd., Carrigtohill, Cork, Ireland). Resulting culture supernatant ultrafiltrate and the filter-sterilized supernatant were stored at −20°C up to 6 months. Supplemented DMEM medium was also ultrafiltrated through a 3 kDa membrane and used as a control.

### Protein profiling of SJPL cells

Sample preparation was performed as described by Auger and collaborators [28]. Briefly, a T25 flask (Sarstedt Inc.) of confluent SJPL cells (0.5 × 10^6^ cells) were infected or not with IAF-Klop PRRSV strain for 4 h (multiplicity of infection (MOI): 0.5) and then treated with AppΔ*apxIC*Δ*apxIIC* culture supernatant or left untreated for 18 h. These conditions are known to block PRRSV replication [7]. Cells were washed twice with Dulbecco's phosphate-buffered saline (DPBS; Life Technologies) and 500 μL of lysis buffer was added (20 mM MOPS (pH 7.0; Sigma-Aldrich), 2 mM EGTA (Sigma-Aldrich), 5 mM EDTA (Fisher Scientific, Fair Lawn, NJ, USA), 30 mM sodium fluoride (Sigma-Aldrich), 60 mM β-glycerophosphate (pH 7.2; Sigma-Aldrich), 20 mM sodium pyrophosphate (Sigma-Aldrich), 1 mM sodium orthovanadate (Sigma-Aldrich), 1 % (v/v) Triton X-100 (Sigma-Aldrich) and one protease inhibitor cocktail tablet Complete Mini EDTA-free (Roche Diagnostics GmbH, Mannheim, Germany), pH 7.2). Cells were removed using a cell scraper (Nalge Nunc International, Rochester, NY, USA) and transferred into a microcentrifuge tube on ice. Sonication was performed at 180 Joules using an ultrasonic processor (Cole-Parmer, Vernin Hills, IL, USA) in order to lyse the cells. Samples were then ultracentrifuged at 90,000 *g* for 30 min in a Sorvall RC M100 ultracentrifuge (Beckman Coulter, Mississauga, ON, Canada). Protein concentration was determined on the supernatant using Pierce BCA protein assay kit (Thermo Fisher Scientific Inc., Rockford, IL, USA) and was adjusted to 2 mg/mL. Samples were then analyzed using Kinex KAM-850 antibody microarray (Kinexus Bioinformatics Corporation, Vancouver, BC, Canada). Kinex KAM-850 antibody microarray includes 854 antibodies which target different cell signaling pathways and included 517 pan-specific antibodies and 337 phosphosite-specific antibodies. These include 466 antibodies against protein kinase, 44 antibodies against protein phosphatase, 46 antibodies against stress response proteins, 75 antibodies against protein implicated in transcription and 223 others. Data were expressed in a chemiluminescence signal ratio versus the untreated cells. The percentage change from control (% CFC) was calculated for each protein by dividing the treated condition Z-ratio by the control Z-scores X 100 as described previously [44].

### Cell cycle analysis

Cell cycle analysis was done using a modified protocol from Sazer and Sherwood [45]. Briefly, 0.5 × 10^6^ SJPL cells were cultured overnight in T75 flask (Sarstedt Inc.) in DMEM. Cells were treated for 18 h at 37 °C in a 5 % CO_2_ atmosphere with either DMEM, the ≤ 3 kDa DMEM ultrafiltrate, AppΔ*apxIC*Δ*apxIIC* undiluted culture supernatant, the ≤ 3 kDa culture supernatant ultrafiltrate, 0.4 μM nocodazole (Sigma-Aldrich), 7.4 μM aphidicolin (Sigma-Aldrich), 100 μM 3,3′-diindolylmethane (DIM; R&D Systems Inc., Minneapolis, MN, USA) or 50 μM SBE-13 hydrochloride (SBE-13; Santa Cruz Biotechnology Inc., Santa Cruz, CA, USA). Cells were washed twice with DPBS and then trypsinized at 37 °C for 10 min. Trypsin (Life Technologies) was inhibited using complete medium and cells were centrifuged at 380 *g* for 15 min at 4 °C. Cells were washed one more time with DPBS and then fixed and permeabilized using 70 % (v/v) cold ethanol for 2 h at room temperature followed by an overnight incubation at 4 °C. Cells were washed twice with DPBS then treated for 2 h at 37 °C with 2 mg of ribonuclease-A (RNase-A; Sigma-Aldrich) in 900 μL of DPBS and cells were finally stained with 100 μL of propidium iodide (PI) 0.2 mg/mL (Sigma-Aldrich). Stained cells were analyzed on BD FACS Calibur (Becton Dickinson, Mississauga, ON, Canada), measuring fluorescence emission at 585 nm after excitation at 488 nm. For each analysis, 10,000 events were evaluated and DNA content was determined by ModFit LT v3.0 (Verity software house Inc., Topsham, ME, USA), which provided histograms to evaluate cell cycle distribution.

### Cell counting

To compare cell proliferation and cell viability during treatments, we used a Countess automated cell counter (Life Technologies). After the trypsinization step in the cell cycle analysis protocol, cells were washed twice in DPBS and centrifuged at 380 *g* for 15 min at 4 °C. Cells were resuspended into 1 mL DPBS and 100 μL of this cell suspension was added to 100 μL of trypan blue (Life Technologies) and 10 μL of this dilution was added to a Countess cell counting chamber slide (Life Technologies) to perform cells count and viability measurement. Data were automatically expressed as number of viable cells per mL.

### Immunofluorescence assay

To detect PRRSV infected cells, we used a modified immunofluorescence assay (IF) protocol from Provost and collaborators which detects PRRSV antigens [39]. Briefly, confluent cells (1 × 10^4^ cells per well in 96-well plates (Corning Inc., Corning, NY, USA)) were infected or not with PRRSV (MOI: 0.5) for 4 h and treated for 44 h with either DMEM, the ≤ 3 kDa DMEM ultrafiltrate, AppΔ*apxIC*Δ*apxIIC* undiluted culture supernatant, the ≤ 3 kDa culture supernatant ultrafiltrate, 100 μM DIM, 0.25 % (v/v) ethanol (DIM solvent), 50 μM SBE-13 or 0.5 % (v/v) H_2_O (SBE-13 solvent). Following infection and/or treatment, cells were washed and then fixed for 15 min at room temperature with a 50 % (v/v) methanol (Fisher Scientific) and 50 % (v/v) acetone (J.T. Baker Inc., Phillipsburg, NJ, USA) solution. Cells were washed three times using phosphate-buffered saline without KCl (PBS): 0.1 M NaCl (Sigma-Aldrich), 4 mM Na_2_HPO_4_ (Fisher Scientific), 1.5 mM KH_2_PO_4_ (Biopharm Inc., Laval, QC, Canada) and then incubated for 90 min at 37 °C with a rabbit monospecific antisera (anti-PRRSV N protein) [43], diluted 1/150 in PBS. Cells were washed three times with PBS and incubated at 37 °C for 60 min with an anti-rabbit antiserum FITC-conjugated (Life Technologies) diluted 1/75 in PBS. Finally, cells were washed three other times with PBS and visualized using a Leica DMI 4000 inverted widefield fluorescence microscope (Leica Microsystems Inc., Richmond Hill, Canada). Pictures were acquired using DFC 490 digital camera (Leica Microsystems Inc.) and images were analyzed using Leica Application Suite Software, version 2.4.0 (Leica Microsystems Inc.).

### Sample preparation for biochemical analysis

In order to eliminate salts and remove inorganic contaminants, a salting-out liquid-liquid extraction was performed. Briefly, 2 mL of ultrafiltrate samples (≤3 kDa) were mixed vigorously for 1 min with 2 g of NaCl (Sigma-Aldrich) and 2 mL of acetonitrile (Fisher Scientific), organic layer was kept after decantation for analysis.

### Thin-layer chromatography

Thin-layer chromatography (TLC) plates were used to analyze samples and preparative TLC plates were used to isolate molecules of interest. For samples analysis, 40 μL of the ≤ 3 kDa ultrafiltrate were applied on a 5 cm × 10 cm (length × width) silica gel plate (Sigma-Aldrich). For preparative TLC plates, 1 mL of the ≤ 3 kDa ultrafiltrates were applied on a 20 cm × 20 cm (length × width) silica gel plate (Sigma-Aldrich). Samples were separated with a mobile phase composed of 75 % (v/v) chloroform (BDH Inc., Toronto, Ontario, Canada) and 25 % (v/v) methanol (Fisher Scientific) in a saturated-closed glass chamber. Migration was performed at ambient temperature (21 °C ± 3 °C). After migration, plates were air-dried for 5 min and then observed under a ultra-violet A (UV-A) light (Ultra-Lum Inc., Claremont, CA). Areas which correspond to spots of interest were scraped from the silica gel plate and transferred into a microcentrifuge tube. The extracted silica was weighed and molecules were extracted with acetonitrile at a ratio of 1:3 (w/v) for 2 h at room temperature. Samples were then centrifuged at 18,000 *g* for 5 min and the supernatant was kept for liquid chromatography tandem mass spectrometry (LC-MS/MS) analysis.

### Instrumentation and bioanalytical methods

The LC-MS/MS system included a Thermo Surveyor autosampler, a Thermo Surveyor MS pump and a Thermo LCQ Advantage Ion Trap Mass Spectrometer (Thermo Scientific, San Jose, CA, USA). Data was acquired and analyzed with Xcalibur 1.4 (Thermo Scientific). Samples were analyzed by LC-MS/MS in full scan positive ion mode. Briefly, 2 μL of sample was injected and the separation was achieved with a gradient mobile phase along with a microbore column Thermo Biobasic C8 (100 × 1 mm) (Thermo Scientific), with a particle size of 5 μm. The initial mobile phase condition consisted of acetonitrile and water (both fortified with 0.4 % (v/v) of formic acid) at a ratio of 5:95. From 0 to 1 min, the ratio was maintained at 5:95. From 1 to 21 min, a linear gradient was applied up to a ratio of 90:10 and maintained for 5 min. The mobile phase composition ratio was then reverted to the initial conditions and the column was allowed to re-equilibrate for 14 min for a total run time of 40 min. The flow rate was fixed at 75 μL/min. Mass spectrometer was coupled with the LC system using a pneumatically assisted electrospray ion source (ESI). Sheath nitrogen gas was set to 10 units and ESI electrode was set to 4000 V in positive mode. Capillary temperature was set at 300 °C and capillary voltage to 34 V. Instrument was operating in full scan MS mode (m/z 200–1000) and MS/MS data was collected on the most abundant peaks in a data dependent acquisition (DDA) mode using a peak intensity threshold of 1 × 10^5^ counts per second (cps). The ≤ 3 kDa ultrafiltrate of DMEM was used as a negative control and spectra were compared with the ≤ 3 kDa ultrafiltrate of the *A. pleuropneumoniae* culture supernatant or the TLC extracted molecules.

### Statistical analyses

For cell cycle data analyses, an arcsine square root ASIN(SQRT(x)) transformation was performed before statistical analysis. When *n* was equal or above eight, a d’Agostino and Pearson omnibus normality test was then utilized, followed by a one-way analysis of variance (ANOVA) with Dunnett post-test or a paired t-test to determine if significant differences exists between the different cell cycle phases or the cell-counting results of untreated and treated cells. All statistical analyses were performed using GraphPad PRISM v5 (GraphPad Software Inc., San Diego, CA, USA). Significant differences were considered when *P* ≤ 0.05.

## Additional file

Additional file 1:
**Protein profiling of SJPL cells after infection or not with PRRSV for 4 h (MOI = 0.5) and/or treatment for 18 h with the App**Δ*apxIC*Δ*apxIIC* **culture supernatant using the Kinex KAM-850 antibody microarray.** (XLSX 48 kb)

## Abbreviations

App: *Actinobacillus pleuropneumoniae*
cps: Counts per second
LC-MS/MS: Liquid chromatography tandem mass spectrometry
m/z: Mass to charge ratio
PRDC: Porcine respiratory disease complex
PRRSV: Porcine reproductive and respiratory syndrome virus
Rf: Retention factor
TIC: Total ion current chromatogram
TLC: Thin-layer chromatography
XIC: Extracted ion chromatogram
